# Impact of the twin pandemics: COVID-19 and oil crash on Saudi exchange index

**DOI:** 10.1371/journal.pone.0268733

**Published:** 2022-05-20

**Authors:** Dania AL-Najjar

**Affiliations:** Finance Department, School of Business, King Faisal University, Al Ahsa, Saudi Arabia; Beihang University, CHINA

## Abstract

This study aims to explore the effects of COVID-19 indicators and the oil price crash on the Saudi Exchange (Tadawul) Trading Volume and Tadawul Index (TASI) for the period from January 1, 2020, to December 2, 2020. The independent variable is oil price, and the COVID-19 indicators are lockdown, first and second decreases of Repo and Reverse Repo rates, Saudi government response, and cumulative deceased cases. The study adopts two phases. In the first phase, linear regression is used to identify the most influential variables affecting Trading volume and TASI. According to the results, the trading volume model is significant with an adjusted R^2^ of 65.5% and a standard error of 81. The findings of this model indicate a positive effect of cumulative deceased cases and first decrease of Repo and Reverse Repo rates and a negative effect of oil prices on Trading Volume. The TASI model is significant with an adjusted R^2^ of 86% and a standard error of 270. The results of this model indicate that lockdown and first decrease of Repo and Reverse Repo rates have a significant negative effect on TASI while the cumulative decrease in cases and oil prices have a positive effect on TASI. In the second phase, linear regression, and neural network predictors (with and without validation) are applied to predict the future TASI values. The neural network model indicates that the neural networks can achieve the best results if all independent variables are used together. By combining the collected results, the study finds that oil price has the most substantial effect on the changes in TASI as compared to the COVID-19 indicators. The results indicate that TASI rapidly follows the changes in oil prices.

## Introduction

COVID-19 is considered one of the worst shocks to have affected all world economies and stopped their major multidimensional activities. The pandemic and the consequent imposition of the lockdown has caused an enormous decline in all economic sectors. Lockdown is one of the most visible and crucial COVID-19 consequences that has caused the deterioration of many macroeconomic factors, oil prices, and the stability and liquidity of stock markets. Hence, many scholars have explored the effects of this pandemic on different sectors and scopes [1–4].

Saudi Arabia is an active and founding member of the United Nations, the Organization of Islamic Cooperation, Gulf Corporation Council, and the Organization of Petroleum Exporting Countries. The Saudi Economy is the largest in the Middle East, the eighteenth largest globally, and is considered a high-income economy based on the definition of the World Bank. Accordingly, the Saudi Economy, Saudi Financial market (Tadawul), and banking sector has attracted the attention of many researchers [4, 5]. Although the Saudi government intervened in the early stages of COVID-19, the Saudi economy faced severe challenges and obstacles in mitigating the effect of the pandemic on macroeconomic factors. As the largest oil exporter globally, the Saudi economy has been facing a difficult period that encompasses the twin pandemics of COVID-19 and the decline in oil prices.

Financial markets are one of the key sectors severely affected during the COVID-19 era, causing stability and liquidity problems in these markets. COVID-19 has pulled down the indices of these financial markets, causing them to him bottom unpredictably, thereby making the prediction of these indices to become even more complicated. The complexity of index prediction already exists because it is the outcome of the interaction of many macroeconomic and international factors together [6–8].

[9] found that the number of confirmed cases is the major indicator that blocked financial activities and caused a decline in the money supply. The findings in [10] revealed that stock markets that are more exposed to China’s economy and have an increasing number of confirmed cases will have a high negative outcome in their indices. Finally, [11] showed that sufficient knowledge, attitude, and practice are success factors for mitigating and regulating COVID-19.

To the best of our knowledge, our paper is the first to study the effects of the oil crash and COVID-19 different indicators on the Saudi Exchange (Tadawul) Index (TASI) and Tadawul trading volume. The key objectives of this study are (i) to identify the effects of each COVID-19 indicator on the TASI and Tadawul trading volume, (ii) to find the effect of oil price crash on TASI and Tadawul trading volume, (iii) to develop prediction models based on neural network and linear regression model for TASI, (iv) to analyze the important parameters based on nonlinear and linear stock prediction models, and (v) to fill the gaps through the most important theoretical background and literature review related to the COVID-19 pandemic.

The study is divided into the following sections. Section Two provides the Literature Review, Section Three discusses the Data, Section Four presents the methodology, Section Five contains the analysis and discussion. Section Six provides the conclusions. Section Seven discusses the theoretical and practical implications, and the limitations and further studies are provided in Section Eight.

## Literature review

This section is divided into five subsections. The first four subsections discuss COVID-19 and stock market indices, trading volume, twin pandemics, and neural network. The fifth subsection considers the recent main attributes of the Saudi economy.

### COVID-19 and stock market indices

Many researchers have investigated the effects of this pandemic on stock market indices and volatility. Their results indicated the existence of a negative significant effect of COVID-19 on stock market indices [12–16]. A positive strong effect of COVID-19 on the stock market volatility was also observed [17–21].

Several researchers have investigated the consequences of this pandemic on developed countries. [22] showed that the effects of COVID-19 differed from one country to another and became less harmful when a country has strong institutional and economic environments. [2, 23] showed that the volatility of US markets is positively related to the increasing number of confirmed and deceased cases. Moreover, [24] found that among the different Taiwanese sectors affected by COVID-19, the manufacturing industry was the worst affected in terms of financial flexibility and return on assets. [25] applied investor attention measured by Google search volume and found that it was negatively correlated to the stock index in G7 and G8 countries. However, [16] investigated the effect of this pandemic on many leading stock markets and identified a negative effect of confirmed cases on stock markets indices.

Researchers have explored the effects of the global pandemic on developing and emerging countries. [26] argued that developing economies were strongly and negatively affected because of the sharp decrease in exports, remittances, and tightening international credit conditions. [27] investigated COVID-19 on regional index return and volatility and determined that Asian markets are stronger than other countries and all regional indices were negatively affected by the pandemic. [28] studied the daily data of 49 countries and found that government intervention was limited in scale and scope. Their findings also revealed that the imposition of lockdown in different sectors worsened the emerging markets’ liquidity. [29] revealed the presence of a negative effect of new and cumulative deceased cases on Jordan, Morocco, and Tunisia stocks markets’ return.

Other scholars compared developed and developing countries in the light of the COVID-19 effect. [30] found that advanced economies through the pandemic era have a strong effect on emerging financial markets, specifically European markets because they transfer the risk to other markets. [31] studied the herding behavior in 72 developed and emerging markets during a pandemic-specific period and found that government response and short selling restrictions alleviated the herding behavior of investors. [32] applied the Barro Misery Index (BMI) to different developed and emerging markets and identified the negative effect of BMI on stock return and a positive effect on stock volatility. The power of this effect differed between developed and emerging markets.

[21] investigated the effects of confirmed and deceased cases on developed and emerging stock markets’ returns and volatility and found that only the confirmed cases have a strong effect on developed markets’ returns, whereas confirmed and deceased cases in emerging markets have a negative effect on the stock market return and a positive effect on market volatility. [14] examined the effects of pandemic indicators on 64 developing and developed countries and determined that stock markets reacted negatively faster to the increase in confirmed cases as compared to the increase in deceased cases. Other scholars investigated the effect of the recent global health pandemic on major Asian financial markets and revealed that abnormal negative return was at a time worse than in the European financial markets [16, 33, 34].

According to [17], financial markets are thought to have bad performance because of COVID-19. As a result, most governments in developed and developing countries imposed a set of actions and formulated many decisive decisions to mitigate the consequences of the pandemic on economies [14, 21, 26, 34–38]. [35] found that increasing the government expenses in general and on education in particular, will make the whole economy have better technological results for the economy. [39] investigated the effect of government response and decisions in addition to other factors on the willingness to use e-wallets in the COVID-19 period and found that this intention differed between countries. [36] found a co-integration relationship of government response and confirmed cases on the stock price of solar enterprises. [40] revealed that having financial contingency plans could protect different businesses in the COVID-19 era.

### COVID-19 and trading volume

Other scholars explored the trading volume before, during, and after the pandemic. Many scholars have studied the effects of the global health pandemic on the trading volume of stock markets. [41] found that the increase in trading activity volume and volatility is associated with staying at home duration. [42] examined the effects of the pandemic on trading volume in different equity markets and showed that trading volume is more intensive with a high willingness to trade when factors, including a high level of trust, wealthy countries, strong legal issues, and a low level of markets’ uncertainty, were present. [21] found that the number of confirmed and deceased COVID-19 cases had a positive effect on the trading volume of equity markets and this result depended on the development classification of the country (i.e. developing or developed). [43] showed that the pandemic was catastrophic in terms of loss in all dimensions, including production, profits, and stock trading.

[44] found that the trading volume before the pandemic had been increasingly fluctuating with an upward trend and experienced a sharp fluctuation with a downward trend during the pandemic. [45] found that no difference in the average abnormal return of medical stocks before and after the declaration of COVID-19 could be observed.

[46] found negative significant abnormal return and trading volume activity in the periods before, during, and after the COVID-19 announcement for Sharia stocks in the capital market in Indonesia. Finally, [47] found that most liquid ETFs have higher trading volume, lower bid-ask spread, higher market capitalization, and lower expenses ratios.

Other researchers investigated the effects of the different types of investors on the trading volume. [48] showed that the abnormal trading volumes of different foreign investors have a significant adverse effect on stock return. [3] revealed the existence of a negative effect of the speculators’ trading on the stability of financial markets. [49] found that retail investors strongly increased trading volume in crisis times.

#### Twin pandemics: COVID-19 and oil crash

The COVID-19 pandemic has continued to cause outcome disruption in all economic spectrums, with oil prices decreasing by more than 20% in one day because of the imposition of lockdown and travel restrictions in most countries. [50] found the existence of a cause-and-effect relationship between COVID-19 and the oil prices and that the oil crash had the strongest effect on the USA stock market.

Many scholars have investigated the effects of the oil crash in parallel with COVID-19 because these twin pandemics create distress and instability in the economies in general and stock markets in particular. [51] found in the long run, oil prices are inversely affected by confirmed cases and COVID-19 had an indirect effect on the oil prices. [52] found that oil prices and the stock market experienced negative short and long-run effects during COVID-19. [53] reported that firms that operate in crude oil and real estate sectors lost more than 70% of the market cap due to COVID-19.

#### Neural network and financial markets

Many academics have reported that the neural network is one of the best models to examine many financial concepts and can be used to explore the effects of COVID-19 on stock financial markets [8, 54–58]. [59] showed that algorithms can predict stock market deterioration. [60] applied the MLP/Neural Network to estimate COVID-19 frequency rates in the US and provided useful insights regarding risk indicators accompanied with COVID-19. [61] applied a multilayer perception algorithm-based model and found that the established artificial neural network (ANN) model can predict and categorize the effects of the COVID-19 outbreak on the welfare of people. [62] further revealed that ANN can estimate the future cases of COVID-19 in different countries. Finally, [57] applied multilayer perception, long-short term memory, convolutional neural network, and one attention-based neural network to predict the next day’s index price according to the historical data. They found that an attention-based neural network has the best performance compared to other models.

#### Saudi economy

Saudi Arabia is a member of the G20 countries with the second-largest petroleum reserves and the fifth-largest natural gas reserves. The Saudi economy is one of the strongest oil-based economies that suffered from the negative consequences of the COVID-19 pandemic and the oil price crash. Since the establishment of OPEC+ to regulate the supply of oil and exert control over its price, oil remained at a price level higher than $50/barrel. The Chinese government imposed a lockdown once the first case of COVID-19 was confirmed in China. Accordingly, China, which is the largest oil importer from Saudi Arabia, decreased its oil demand, which caused a serious economic shock for the Saudi economy. The consequences of COVID-19 on Saudi economies hit many pillars, starting from Fitch and Moody’s agency, which downgraded the outlook of the Saudi economy. IMF and World Bank found the Saudi GDP growth to be -5.4% and -3.8%, respectively in 2020 because of COVID-19 and its consequences. Most of the macroeconomic indicators also showed a significant effect since the start of the pandemic, with the unemployment rate increasing to around 15.4%, and TASI reaching the bottom with a 29.7% drop in 2020.

The Ministry of Finance issued the “Saudi budget statement for 2021” and showed that GDP growth is estimated to be -3.7% in 2020 and 3.2% in 2021. The Saudi budget deficit increased to around SAR 298 billion at end of 2020 and is expected to be SAR 141 billion at end of 2021. Public debt is around SAR 854 billion in 2020 (representing 34.3% of the estimated GDP) and is expected to be SAR 937 billion in 2021 (around 32.7% of the expected GDP). Finally, government reserves at Saudi Central Bank (SAMA) are estimated to be SAR 346 billion in 2020 and expected to be SAR 280 billion in 2021.

After the first COVID-19 cases were confirmed, the Saudi government and Saudi Central Bank (SAMA) announced a set of support packages aimed to assist the private sector and included exemptions and rescheduling of some government dues and wage subsidy of 60% of Saudi employees’ salaries in the private sector. The SAMA injected a huge amount (around $13 billion) into the banking sector to continue providing facilities to the private sector.

According to [63] COVID-19 had an inverse effect on TASI. [64] studied the effects of oil price on the growth of GCC economies and revealed the positive effect of oil price on Saudi economic growth, a negative effect in Kuwait and Qatar with no significant effect of oil price on Bahraini and Omani economic growth. [65] investigated the key oil-producing and oil-consuming countries and found a significant interdependence between these countries.

In summary, many researchers have investigated the effects of COVID-19 on stock market indices and volatility in both developing and developed countries. Their results highlighted the negative effect of COVID-19 on stock market indices [1, 12–16, 26, 27, 29, 32] and a positive effect on stock market volatility [17–21].

Other scholars explored the effect of COVID-19 indicators and stock liquidity on trading volume through the COVID-19 era. Scholars found that confirmed and deceased COVID-19 cases positively affected the trading volume of equity markets. This result depends on the development classification of the country (i.e., developing or developed). However, few scholars examined the effect of the oil crash in parallel with COVID-19 on financial markets indices [51, 52] in general and the effect of COVID-19 and oil prices on TASI in specific [4, 64, 65].

Several researchers investigated the consequences of confirmed and deceased cases on the financial market indices of developed and developing countries [2, 14, 21, 23, 29]. Other researchers have also explored the effect of imposing a set of actions and formulated many decisive decisions to mitigate the consequences of the pandemic on economies [14, 21, 26, 34–39].

Finally, many academics have reported that a neural network is one of the best models to examine many financial concepts and can be used to explore the effects of COVID-19 on financial stock markets [8, 54–58].

Accordingly, our research questions are as follows:

1. What are the determinants of the TASI and Tadawul trading volume during the COVID-19 period?
2. Did the oil crash affect the TASI and Tadawul trading volume during the COVID-19 period?
3. Can we build a neural network model to predict the TASI?
4. What are the main parameters that can predict the TASI?

## Research methodology

This section is divided into three subsections including data collection, data analysis, and development of stock market prediction.

### Data collection

In this paper, we investigate the effects of the global pandemic indicators on the TASI and Tadawul trading volume, from January 1 to December 2, 2020. The COVID-19 indicators are lockdown, first decrease and second decrease of Repo and Reverse Repo, Saudi government response, cumulative deceased cases, and oil prices. The sources of data are John Hopkins, Tadawul, and the Saudi Central Bank (SAMA) websites.

In this paper, TASI refers to the daily level of the Saudi Exchange (Tadawul) index. Trading volume is the number of stocks traded daily in the Saudi Exchange (Tadawul). Both dependent variables are lagged for one period, which is one day.

A lockdown was imposed in Saudi Arabia on March 9, 2020. The government imposed a 24-hour curfew and nationwide lockdown to address the COVID-19 pandemic and minimize the number of confirmed cases. People were not allowed to move freely and were only allowed to get their basic needs, such as food until 3 pm. Fines were imposed on anyone who did not obey these regulations. Saudi regulatory authorities urged all citizens and residents to comply with the safety issues, especially of the social distancing policy. Consequently, most of the activities and workplaces of the economy were blocked. In this study, lockdown as one of the COVID-19 indicators will be used to investigate its effect on Tadawul trading volume and TASI [1, 2, 63]. Lockdown is measured by date because it was imposed on March 9, 2020.

Repo and Reverse repo rates are the rates used by central banks to control inflation and money supply. Repo is the rate at which the central banks lend money to commercial banks when faced with a funds shortage.

The Reverse Repo rate is the opposite of the repo in which the central bank of the country borrows money from commercial banks. The decreasing rates of Repo and Reverse Repo are the main and fastest action that the Saudi Central Bank (SAMA) took to mitigate the consequence of COVID-19. This decrease was applied in two phases; the first decrease was 50 points and the second decrease was 75 points in both rates. This action was first applied by the US Federal Reserve to maintain and enhance the stability and growth levels of the economy during the pandemic [38]. We measured the first and second decreases of the Repo and Reverse repo rates as dates because they were imposed on March 3 and 16, 2020, respectively.

The government’s response was crucial to support all sectors through the pandemic period. The Saudi government’s response was applied to mitigate the effects of the pandemic. It involves imposing regulations and support packages as increasing the external borrowings and injecting extra money to support private businesses in many aspects. The Saudi government’s response is one of our independent variables, and we intend to determine its effect on TASI and Tadawul trading volume [34, 37, 38]. We measured the Saudi Government response as a date variable starting from the 14^th^ of March.

According to the Saudi Ministry of Health, the first confirmed COVID-19 case appears on March second^,^ while the first deceased case was on March 22. Cumulative deceased COVID-19 cases are implemented as an independent variable in our study [14, 16, 21, 29].

The oil price crash is one of the COVID-19 indirect consequences that affected all countries and is considered one of the most important factors that affected most of the economies worldwide. The Saudi economy depends deeply on oil revenues because Saudi Arabia is an oil-based economy. Several scholars studied the effect of the oil prices crash on stock market indices and Saudi economic growth [50, 64, 65]. In this study, we used Crude Oil WTI Future for oil prices in Tadawul trading volume and TASI models.

### Data analysis and linear regression models

According to our methodology, we will run a linear regression model to specify the drivers of the TASI and Tadawul trading volume using different removing and adding variables methods. The statistical analysis of the collected dataset is shown in Table 1, which presents the main COVID-19 indicators, oil prices, TASI, and Tadawul trading volume for the period from January 1 to December 2, 2020. Table 1 shows that there were 230 observations for the mentioned period and that the cumulative deceased cases on average for the whole period is 2205 cases. Moreover, oil prices fluctuated through the COVID-19 period, and on average, it was $31 with a maximum price of $64.9. According to the average TASI and trading volume, TASI has a maximum of 8,747 and on average was 7,642. The trading volume has a maximum of 782 million stocks and on average was 302 million stocks for the period from January 1 to December 2, 2020.

**Table 1:** Descriptive statistics of the independent and dependent variables.

	N	Minimum	Maximum	Mean	Std. Deviation
**Saudi Government Response**	230	0	1	0.7565	0.43012
**First decrease rate**	230	0	1	0.0435	0.20438
**Second decrease rate**	230	0	1	0.7696	0.42203
**Cumulative deceased cases**	230	0	5930	2097.574	2205.657
**Oil price**	230	7.79	64.92	31.307	15.26939
**Lockdown**	230	0	1	0.3435	0.47591
**TASI**	230	5959.69	8747.09	7642.949	710.5164
**Trading Volume (MM of stocks)**	230	96.87	782.26	302.63	139.0126

The dataset shows the TASI and Tadawul trading volumes are presented as dependent variables with COVID-19 indicators (lockdown, first decrease of Repo and Reverse Repo and second decrease of Repo and Reverse Repo, Saudi government response, cumulative deceased cases, and oil prices) as independent variables.

Pearson’s correlation is applied to determine the relationship between the independent and dependent variables as shown in Table 2. The results showed that trading volume has a significant positive relationship with month, Saudi government response, cumulative deceased cases, and second decrease of Repo and Reverse Repo rates. However, trading volume has a significant negative relationship with lockdown and an insignificant negative relationship with oil prices.

**Table 2:** Descriptive statistics of main variables.

	Gov. Response	first Decrease of rates	second Decrease of rates	Cumulative deceased	Oil prices	lockdown	Trading Vol.	TASI
**Gov. response**	**1**	**-0.376**	**0.965**	**0.541**	**-0.673**	**0.24**	**0.508**	**-0.09**
**Sig.**	** **	**0**	**0**	**0**	**0**	**0**	**0**	**0.173**
**First decrease of rates**	**-0.376**	**1**	**-0.39**	**-0.203**	**0.07**	**0.07**	**-0.08**	**-0.265**
**Sig.**	**0**	** **	**0**	**0.002**	**0.293**	**0.289**	**0.229**	**0**
**Second Decrease of rates**	**0.965**	**-0.39**	**1**	**0.522**	**-0.698**	**0.287**	**0.506**	**-0.156**
**Sig.**	**0**	**0**	** **	**0**	**0**	**0**	**0**	**0.018**
**Cumulative deceased**	**0.541**	**-0.203**	**0.522**	**1**	**0.131**	**-0.545**	**0.777**	**0.644**
**Sig.**	**0**	**0.002**	**0**	** **	**0.048**	**0**	**0**	**0**
**Oil prices**	**-0.673**	**0.07**	**-0.698**	**0.131**	**1**	**-0.69**	**-0.015**	**0.701**
**Sig.**	**0**	**0.293**	**0**	**0.048**	** **	**0**	**0.823**	**0**
**lockdown**	**.240** ^**^	**0.07**	**.287** ^**^	**-0.545**	**-0.69**	**1**	**-0.339**	**-0.812**
**Sig.**	**0**	**0.289**	**0**	**0**	**0**	** **	**0**	**0**
**Trading Vol.**	**0.508**	**-0.08**	**0.506**	**0.777**	**-0.015**	**-0.339**	**1**	**0.463**
**Sig.**	**0**	**0.229**	**0**	**0**	**0.823**	**0**	** **	**0**
**TASI**	**-0.09**	**-0.265**	**-.156** ^*^	**0.644**	**0.701**	**-0.812**	**0.463**	**1**
**Sig.**	**0.173**	**0**	**0.018**	**0**	**0**	**0**	**0**	** **

The outcomes of the correlation matrix showed that TASI is not significant with Saudi government response, and TASI had a significant negative weak relationship with first decrease and second decrease rates. In addition, TASI had a significant positive relationship with the month and cumulative deceased cases. The results also indicated that TASI has strong positive and negative relationships with the oil price and lockdown, respectively.

The results of correlation indicate that multicollinearity can be observed in the independent variables. Therefore, to solve the multicollinearity problem, a variable inflation factor (VIF) is used. The VIF is used to drop the variable(s) with a VIF of more than 10 and keep the variable(s) with a VIF of less than 10. Accordingly, VIF stepwise variable selection method is applied to choose the model with independent variables with a VIF of less than 10.

In the trading volume model, the results revealed that the model, which consists of cumulative deceased cases, oil price, and the first decrease in Repo and Reverse Repo, can pass the multicollinearity test as shown in Table 3.

**Table 3:** Variable inflation factor analysis with Trading Volume as a dependent variable after dropping highest VIF.

	Collinearity Statistics	
Variables	Tolerance	VIF
**Oil price**	.977	1.024
**First decrease in Repo and Reverse Repo**	.955	1.048
**Cumulative deceased cases**	.938	1.066

The results showed that if the month, second decrease in Repo and Reverse Repo, and Saudi government response are dropped from the analysis of the TASI model, the model can pass the multicollinearity test as shown in Table 4.

**Table 4:** Variable inflation factor analysis with TASI as dependent variable after dropping highest VIF.

	Collinearity Statistics	
Variables	Tolerance	VIF
**Oil price**	.435	2.297
**Lockdown**	.313	3.196
**first decrease in Repo and Reverse Repo**	.920	1.086
**Cumulative deceased cases**	.574	1.741
**Day**	.961	1.040

Finally, the study will build two linear regression models using the solved multicollinearity models. The first model uses the Tadawul trading volume and the second model uses TASI as the dependent variable. A linear regression predictor will be built and compared to the neural network model as discussed in the following section.

### Development of stock market prediction models

**Fig 1:**
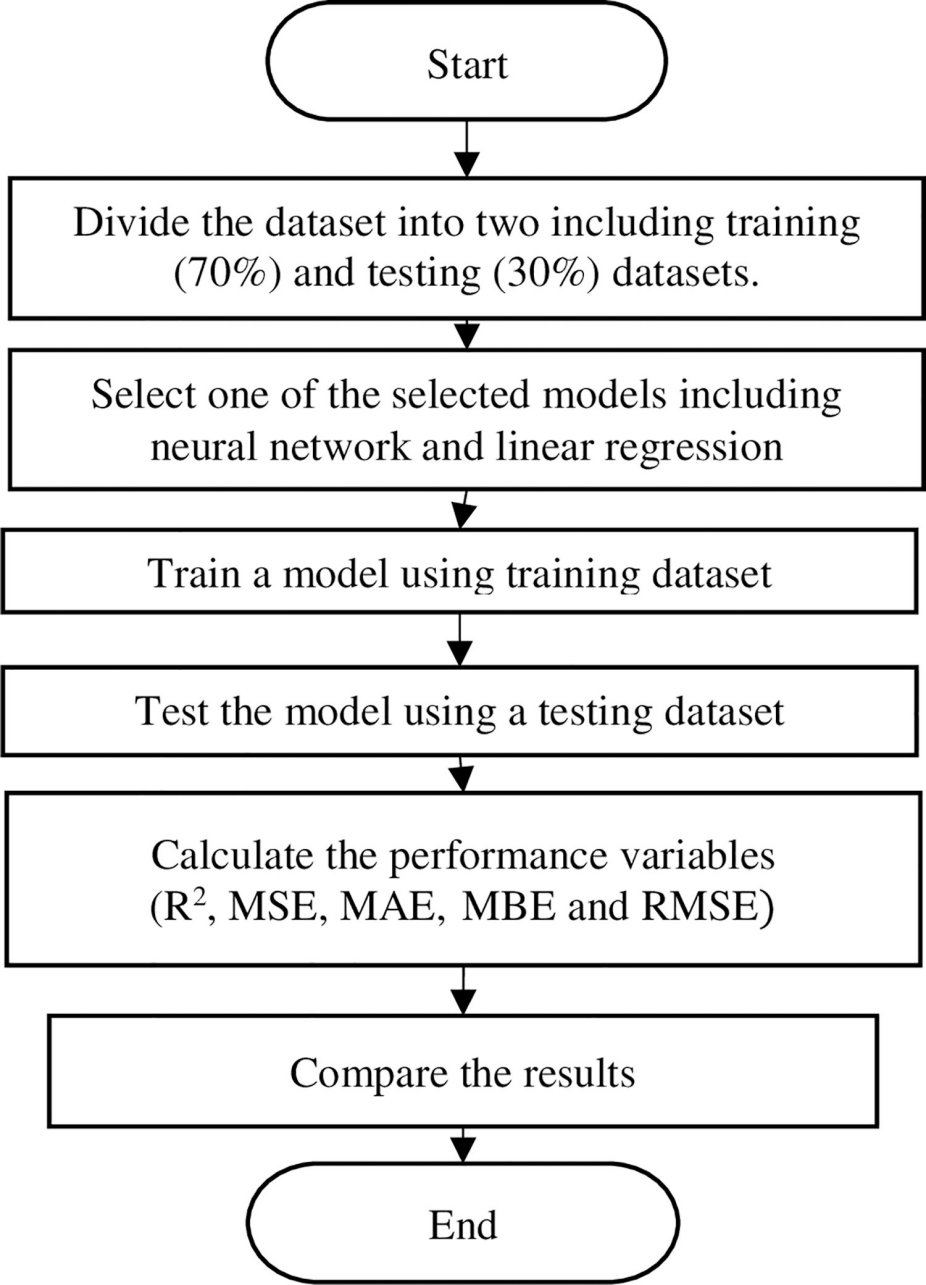
Flowchart of neural network and linear regression prediction model.

After building the volume and stock market regression model using linear regression, the collected data of the stock market will be reprocessed using months and time with the previous inputs to build prediction models [8, 58, 66–71]. The study adopted two prediction models to calculate the predictor, including linear regression and neural network. Before building the prediction models, the collected dataset will be divided into training, testing, and validating datasets. After testing the trained models, the predictor importance variables will be compared with the collected results from the regression model. The data set partition is retested by repartitioning the dataset into three datasets to optimize the trained neural network. Data sets are trained, tested, and validated. The goal of dataset validation is to improve the ability of the neural network to accurately predict future cases and improve the biases and weights of the trained neural network. The flowchart of the applied methodology is shown in Fig 1.

When building a prediction model using a neural network and linear regression model, some parameters must be tuned to achieve a high prediction model as shown in Tables 5 and 6.

**Table 5:** Parameters for the neural network.

Parameter	Value
**Number of Epochs**	1000
**Mu**	1 x 10^10^
**Gradient Descent Value**	1 x 10^−7^
**Performance**	1 x 10^−15^
**Total number of parameters**	9
**Training ratio**	70%
**Testing ratio**	30% (15% in case of validation)
**Validation ratio**	15%
**Number of neurons (hidden layer/s)**	6
**Optimization Algorithm**	Gradient decent
**Maximum Training Time**	15 minutes

**Table 6:** Parameters for linear regression.

Parameter	Value
**Selection Method**	Forward Stepwise
**Entry for entry/removal**	Information criteria (Hurvich and Tsai’s Criterion)
**Total number of parameters**	9
**Training ratio**	70%
**Testing ratio**	30%
**Maximum Training Time**	15 minutes

Six inputs are considered in building a prediction model: Lockdown, Cumulative deceased cases, Oil Price, First decrease in Repo and Reverse Repo, Second decrease in Repo and Reverse Repo, and Saudi Government response.

### Performance metrics

The performance of the prediction models can be evaluated using various metrics. In this study, determination coefficients (R^2^) and four error functions are used. The four error functions are mean absolute error (MAE), root mean square error (RMSE), mean bias error (MBE), and mean square error (MSE) [8, 69]

$$R^{2}(determination\;coefficient)=1-\frac{\sum_{i=1}^{N}{\left(y_{i}-\hat{y_{i}}\right)}^{2}}{\sum_{i=1}^{N}{\left(y_{i}-\bar{y}\right)}^{2}}$$ (1)

$$MAE=\frac{1}{M}\sum_{i=0}^{N}|y_{i}-\hat{y_{i}}|$$ (2)

$$RMSE=\sqrt{\frac{1}{M}\sum_{i=0}^{N}{\left(y_{i}-\hat{y_{i}}\right)}^{2}}$$ (3)

$$MBE=\frac{1}{M}\sum_{i=0}^{N}y_{i}-\hat{y_{i}}$$ (4)

$$MSE=\frac{1}{M}\sum_{i=0}^{N}{\left(y_{i}-\hat{y_{i}}\right)}^{2}$$ (5)

where $y_{i},\;\hat{y_{i}}$, and $\bar{y}$ are the real stock market, the predicted stock market, and the mean of the stock market, respectively. In addition, M and i are the number of samples and the index of the data in the dataset, respectively.

## Analysis and results discussion

The analysis will be applied using Tadawul trading volume as the first model, then TASI, and the TASI Prediction Model.

### Results of Tadawul trading volume

In this section, the stock market regression model will be constructed to explore the important factors that have influenced the trading volume. Tables 7 and 8 show that the model is significant with an adjusted R^2^ of 65.5% and a standard error of 81.64.

**Table 7:** Model summary.

Model	R	R^2^	Adjusted R^2^	Std. Error of the Estimate
**1**	0.812^a^	0.66	0.655	81.644

a. Predictors: (Constant), cumulative deceased cases, oil price, first decrease in Repo and Reverse Repo.

**Table 8:** ANOVA a test of the linear regression model.

Model	Sum of Squares	Df	Mean Square	F	Sig.
**Regression**	2918,868.172	3	972956.057	145.965	.000b
**Residual**	1506440.104	226	6665.664		
**Total**	4425308.276	229			

Table 9 shows that the cumulative deceased cases, oil prices, and the first decrease in Repo and Reverse Repo are the significant variables that affect trading volume.

**Table 9:** Coefficients of the reduced linear regression model without multicollinearity.

Model	Unstandardized Coefficients		Standardized Coefficients	t	Sig.
	B	Std. Error	Beta		
**(Constant)**	228.125	12.909		17.672	.000
**Cumulative deceased cases**	.053	.003	.834	20.823	.000
**Oil price**	-1.205	.358	-.132	-3.370	.001
**First decrease in Repo and Reverse Repo**	63.903	27.019	.094	2.365	.019

a. Dependent Variable: Trading Volume.

Both first decreases in Repo and Reverse Repo rates and cumulative deceased cases have a significant positive effect on Tadawul’s trading volume. The decreased interest rate was applied to mitigate the negative consequences of the pandemic. However, cutting the interest rates led to a decrease in Tadawul trading volume. Theoretically, decreasing interest rates enhance the tendency of investors to seek extra money and increase their investments, which in turn will increase the trading volume. The same observation can be seen for the cumulative cases, indicating a positive effect on the trading volume of TASI. However, the current situation of the COVID-19 pandemic and lockdown situation resulted in the investors having no other choices to invest their money in other than financial markets. Different media channels were also not focused on the number of deceased cases as confirmed cases. This improvement in trading volume can also be because of the serious and quick actions to mitigate the effects of COVID-19 on all economic sectors. These actions reduced the spread of the pandemic in general and enhanced the confidence of the financial system in KSA. Future studies can apply other macroeconomic factors to explore the effects of the pandemic on the financial market indices.

These results are consistent with the findings of [21] in those cumulative deceased cases positively affect the trading volume, and [42] found that trading volume is positively related to strong economies that have a high level of trust and lesser market uncertainty. While this result contrasts with [43, 44, 46] as their findings showed a negative effect of COVID-19 on trading volume.

Oil prices had a negative effect on trading volume, that is, trading volume increased because of the sharp decrease in oil prices during the pandemic period, that is, through the twin pandemics of COVID-19 and the oil crash; there were many factors play together to increase the confidence in the Saudi economy and stock market. Consequently, more investors were directing their investments from different types of investments and injecting more into Tadawul. This result is in line with the findings of [41] that showed increasing trading volume during the lockdown period.

### Results of TASI regression model

In this section, the regression model will be constructed to explore the important factors that have influenced TASI. Tables 10 and 11 show that the model is significant with an adjusted R^2^ of 86% and a standard error of 270.

**Table 10:** Model summary.

Model	R	R^2^	Adjusted R^2^	Std. Error of the Estimate
1	0.926^a^	0.86	0.86	270.88

a. Predictors: (Constant), Day, Lockdown, First decrease in Repo and Reverse Repo, Cumulative deceased cases, Oil price.

**Table 11:** ANOVA test of the linear regression model.

Model	Sum of Squares	Df	Mean Square	F	Sig.
**Regression**	99169894	5	19833979	270	.000
**Residual**	16436995	224	73379		
**Total**	115606889	229			

Table 12 shows that lockdown has a significant negative effect on TASI. Lockdown is the first observable consequence that accompanied this pandemic. It blocked all activities on all levels, including people, companies, and the stock markets. This result is in line with [1, 2, 25, 32, 53].

**Table 12:** Coefficients of the reduced linear regression model without multicollinearity.

B			Standardized Coefficients	T	Sig.
		Std. Error	Beta		
**(Constant)**	6806.363	94.099		72.332	.000
**Oil_price**	23.656	1.777	.508	13.314	.000
**Lockdown**	-324.048	67.241	-.217	-4.819	.000
**first decrease in Repo and Reverse Repo**	-716.298	91.294	-.206	-7.846	.000
**Cumulative deceased cases**	.135	.011	.420	12.633	.000

Dependent Variable: TASI.

The findings of this paper indicates that the cumulative deceased cases have a significant positive effect on TASI. As mentioned previously, the enhancement of trading volume through the pandemic era was the result of a mix of many interrelated factors related to the imposition of special regulations and the improvement of health services. These vital actions successfully addressed the pandemic and slowed down the marginal increase in the number of deceased cases. This result is in contrast with [21, 29], who found a negative effect of new and cumulative deceased cases on stock returns.

Oil prices reflect what is happening in the world economy and are an important indicator that will direct the future of many industries. Oil prices also influence the costs of other productions and manufacturing all around the world, and COVID-19 led to strong fluctuations in oil prices worldwide. Saudi Arabia, as an oil-based economy with one of the highest oil reserves in the whole world, was strongly affected by the oil crash because oil revenues are the main source of revenue in the Saudi economy. Table 12 shows the existence of a positive significant effect of oil prices on TASI. This result is in line with [50, 52, 64, 65].

The central bank of Saudi Arabia decreased Repo and Reverse Repo with a total of 125 points because of COVID-19. Government intervention in the form of decreased rates aimed to enhance the economic growth of the Saudi economy. Decreasing interest rates is one of the government responses during the pandemic because interest rate decreases in the Saudi economy cause the fixed income instruments and deposits to no longer be attractive to investors. Accordingly, investors decided to transfer their investments from these types of investments that give low returns to the stock market (Tadawul), which may offer higher return rates. According to the analysis, the decreases in Repo and Reverse Repo have a significant negative effect on TASI. This result contrasts with [37, 38].

Moving to the lockdown variable, the reactions of the Saudi government were levied from the Saudi Central bank and Saudi government through different fiscal and monetary tools that affect most of the sectors and enhance the growth of the Saudi economy. Lockdown is the first observable consequence that accompanied this pandemic; it blocked all the activities on all levels, including people, companies, and the stock markets. Accordingly, most of the sectors and Tadawul deteriorated sharply because of this decision. The lockdown variable is one of the COVID-19 indicators and as shown in Table 12, the lockdown has a significant negative effect on TASI. This result is in line with [1, 2, 25, 34, 53].

COVID-19 indicators and oil prices have a strong effect on TASI and trading volume, and some of these indicators have a positive effect and others have a negative effect. In general, most of the COVID-19 indicators have a strong effect on TASI, which is consistent with several studies such as [1, 12–15, 37, 63].

Finally, the analysis indicated that oil price, lockdown, the first decrease in Repo and Reverse Repo rates, and cumulative deceased cases were the most dominant and effective factors on TASI. The suggested model will be used as a linear predictor to develop a prediction model that can be used to predict future TASI values.

## Robustness test with control variable

This section will rerun the two models of trading volume and TASI using interest rates as the control variables. In these models, we will add the value of interest rate variables (i.e., Repo and Re-repo rate) as control variables, then rerun these two models.

### Trading volume model

Several researchers used macroeconomic indicators as control variables. The author will add the control variables in this section, as shown in Tables 13–15 below, using trading volume as the dependent variable.

**Table 13:** Model summary.

Model	R	R Square	Adjusted R Square	Std. Error of the Estimate
**1**	0.814^a^	.662	.658	81.30728

a Predictors: (Constant), Cumulative Deceased Cases., Repo Rate, FirstDecRate.

**Table 14:** ANOVA.

Model	Sum of Squares	df	Mean Square	F	Sig.
**Regression**	2931250.892	3	977083.631	147.799	.000
**Residual**	1494057.384	226	6610.873		
**Total**	4425308.276	229			

**Table 15:** Coefficients of trading volume model with control variable.

Model	Unstandardized Coefficients		Standardized Coefficients	t	Sig.	Collinearity Statistics	
	B	Std. Error	Beta			Tolerance	VIF
**(Constant)**	261.374	20.179		12.953	.000		
**Cumulative Deceased Cases.**	.046	.003	.733	16.199	.000	.729	1.372
**Repo Rate**	-45.573	12.486	-.165	-3.650	.000	.732	1.367
**FirstDecRate**	68.021	26.996	.100	2.520	.012	.948	1.054

a. Dependent Variable: Volume.

As presented in the tables above, the new model showed significance and high adjusted R-squared after adding the control variables. Both trading volume models (i.e., with and without control variables) have almost the same adjusted R-squared and standard error of the estimates (i.e., 0.658 and 81.3 VS 0.655 and 81.6, respectively). Also, both models showed that cumulative deceased cases and the first decrease in interest rate positively affect trading volume. Besides, the new model showed that the Repo rate (i.e., the control variable) has a significant negative effect on trading volume.

### TASI model

In this part, the author will rerun the TASI model using control variables (i.e., the value of Repo and Re-repo rate). As presented in Tables 16–18 below, the new model showed significance and high adjusted R-squared after adding the control variables. Both TASI models (i.e., with and without control variables) almost have the same adjusted R-squared and standard error. Also, both models showed that cumulative deceased cases and oil price positively affect TASI. The first decrease in interest rate showed a negative effect on TASI. Besides, the new model showed that the Re-repo rate (i.e the control variable) has a significant effect on TASI.

**Table 16:** Model summary.

Model	R	R Square	Adjusted R Square	Std. An error in the Estimate
**1**	0.941^a^	.886	.884	242.42080

a. Predictors: (Constant), Cumulative Deceased Cases., Rerepo_Rate, FirstDecRate, OilPrice.

**Table 17:** ANOVA.

Model	Sum of Squares	df	Mean Square	F	Sig.
**Regression**	102384123.801	4	25596030.950	435.545	0
**Residual**	13222764.858	225	58767.844		
**Total**	115606888.659	229			

**Table 18:** Coefficients of TASI model with control variable.

Model	Unstandardized Coefficients		Standardized Coefficients	t	Sig.	Collinearity Statistics	
	B	Std. Error	Beta			Tolerance	VIF
**(Constant)**	6208.883	44.877		138.354	.000		
**Cumulative Deceased Cases.**	.287	.016	.886	17.409	.000	.196	5.098
**Rerepo_Rate**	861.466	104.346	.610	8.256	.000	.093	9.739
**FirstDecRate**	-880.470	80.766	-.253	-10.902	.000	.942	1.062
**OilPrice**	6.842	2.976	.147	2.299	.022	.124	8.045

a. Dependent Variable: TASI.

### Results of TASI prediction models

**Fig 2:**
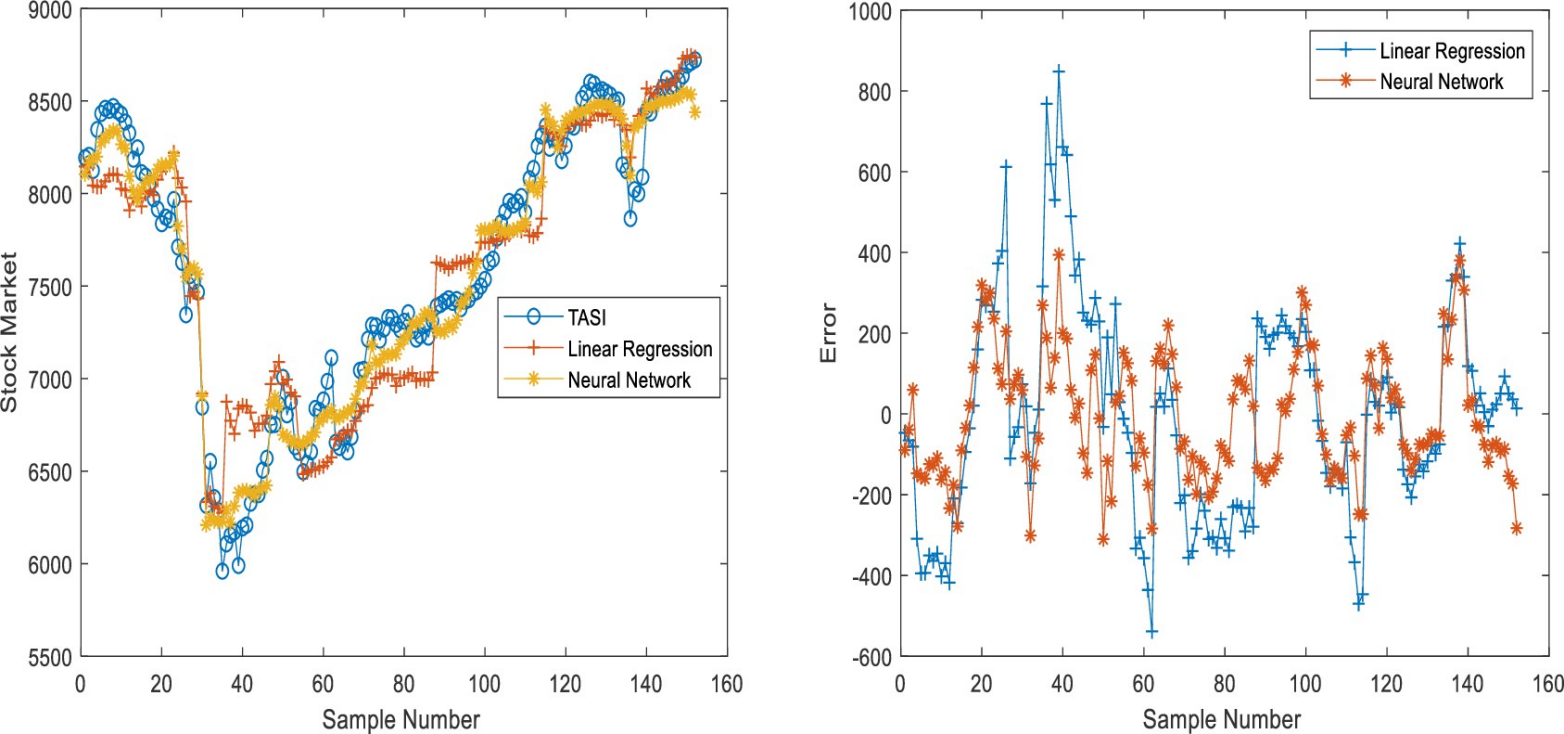
Performance analysis of training datasets using (a) predicted values and (b) Error functions based on neural network and linear regression.

**Fig 3:**
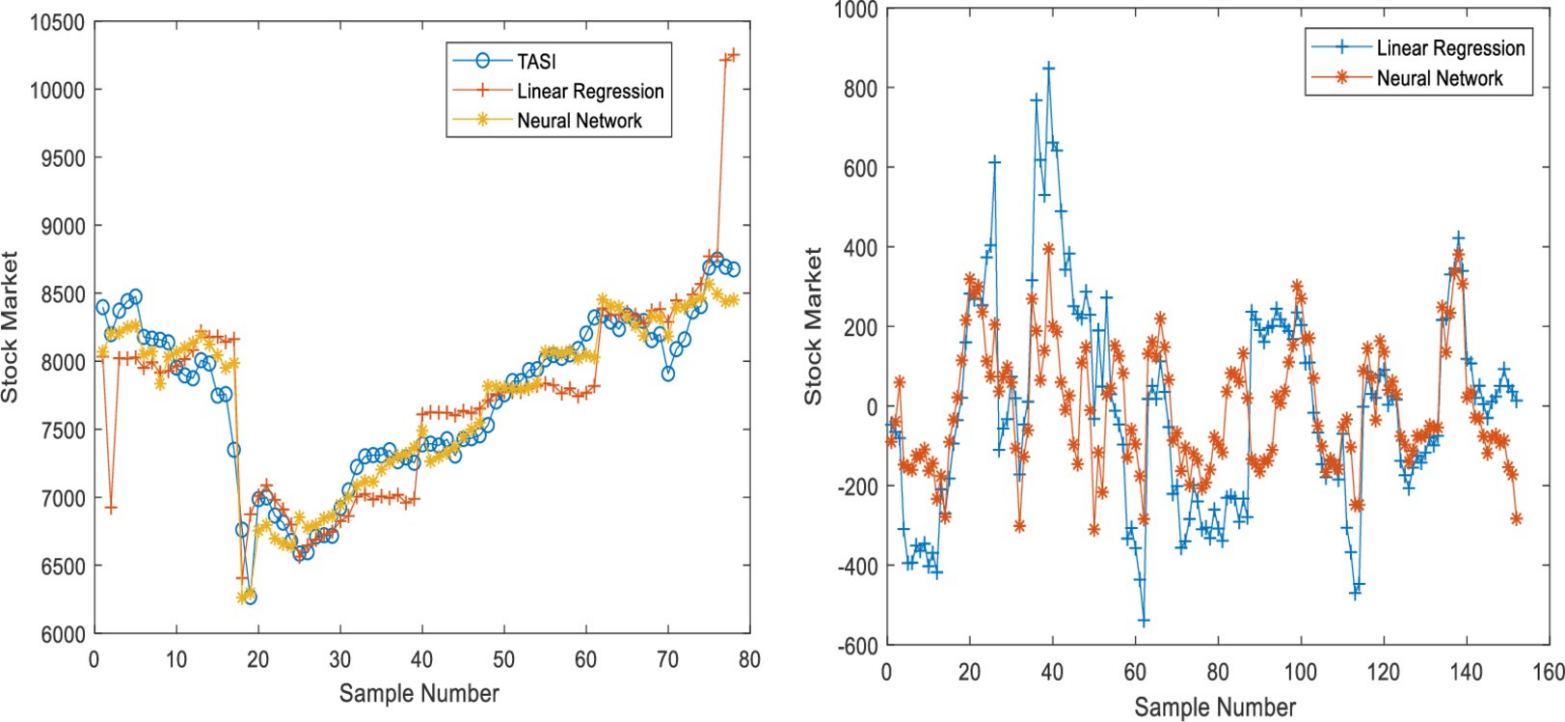
Performance analysis of testing datasets using (a) predicted values and (b) Error functions based on neural network and linear regression.

**Fig 4:**
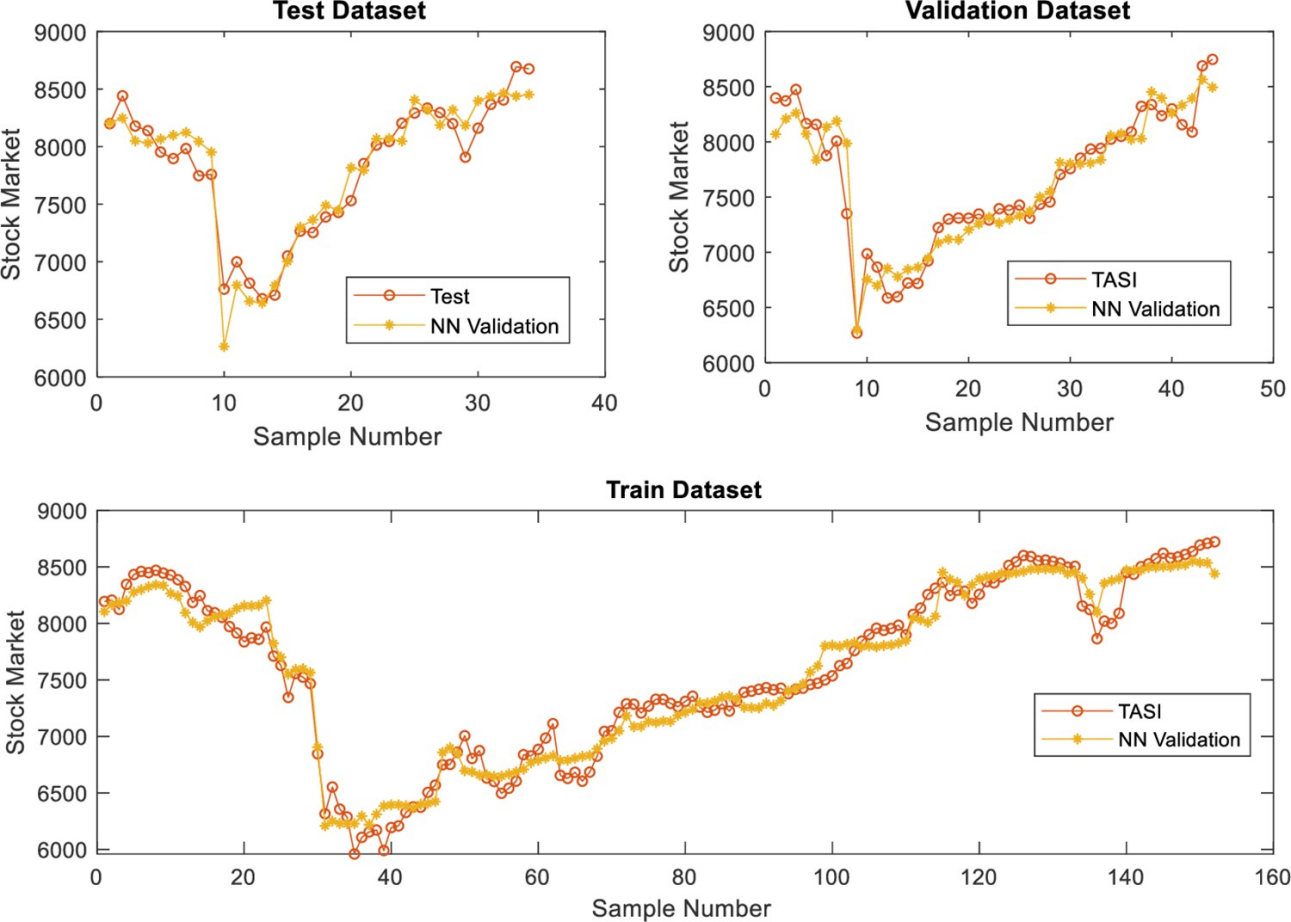
Performance analysis of neural network based on the validation dataset.

**Fig 5:**
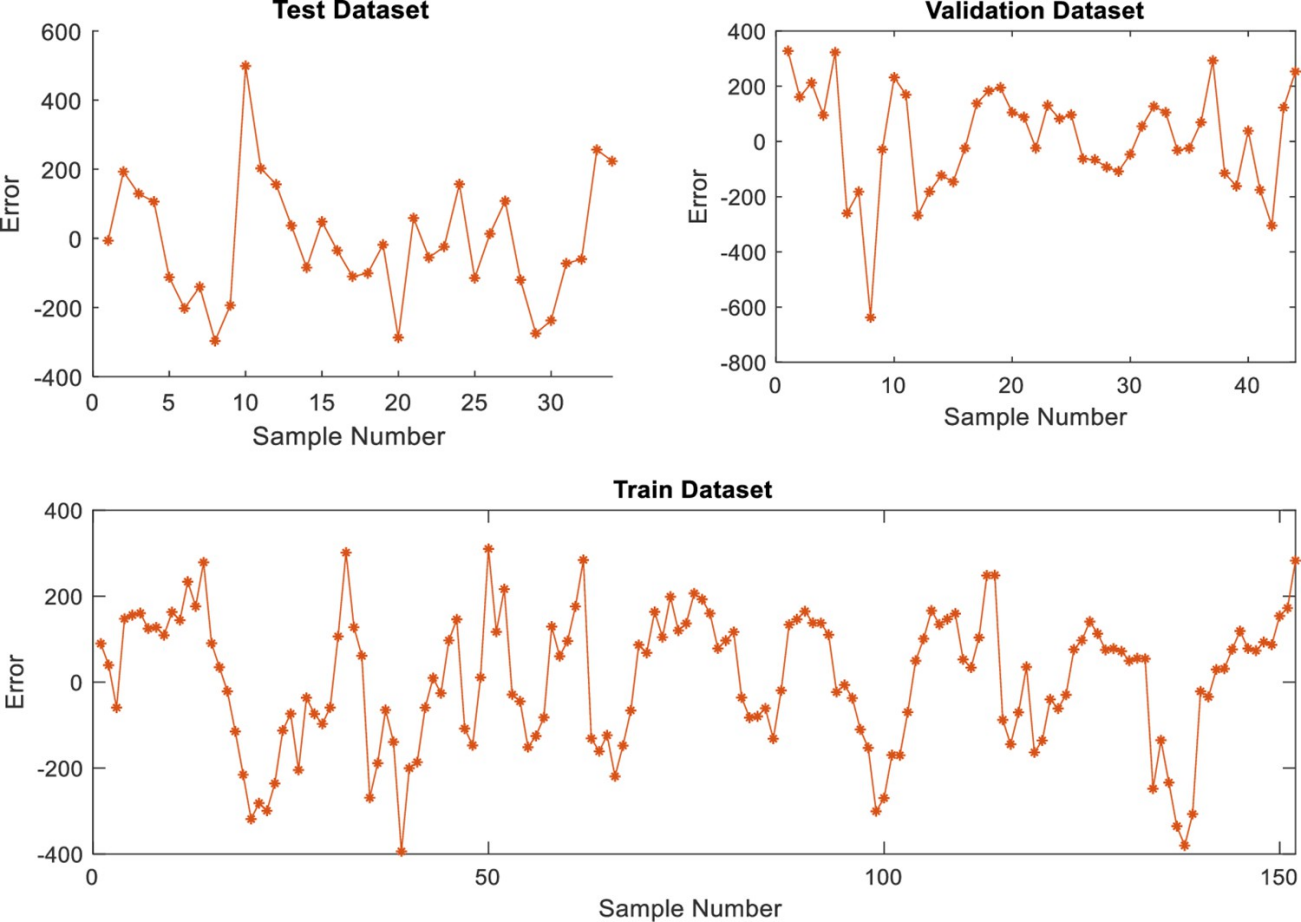
Error analysis of neural network based on the validation dataset.

The predicted and real values are plotted to follow the gaps between the predicted and real values and interpret the performance of the stock market predictors based on linear regression and neural networks (with two cases). The error functions and determination coefficients of the trained and tested datasets are also used. Figs 2–5 show the performance based on linear regression, neural network without validation (NN), and neural network with validation (NN validation) of the training, testing, and validation datasets, respectively. The results showed that the neural networks follow the orientation of the real values compared to the predicted linear regression model. The neural network model with two developed models (NN and NN validation) also had lower error attenuation for both data sets, including testing, training, and validation.

The collected results from graphs do not show the real efficiency of the proposed prediction models, and therefore, the four error functions and determination coefficient are used with both predictors. The results of the training dataset using linear regression model are 0.94, 71485, 211, 0.00, and 267 for R^2^, MSE, MAE, MBE, and RMSE, respectively, for testing dataset using linear regression model, the results are 0.85, 145897, 256, -17.00, and 382 for R^2^, MSE, MAE, MBE, and RMSE, respectively as shown in Table 19. The results of the training dataset using neural network model are 0.98, 23620, 130, 6.22, and 154 for R^2^, MSE, MAE, MBE, and RMSE, respectively, whereas for a testing dataset using neural network model, the results are 0.95, 33176, 146, 2.23 and 182 for R^2^, MSE, MAE, MBE, and RMSE, respectively as shown in Table 19. The training results of neural network validation are 0.98, 23620, 130.16, 6.22, and 154 for R^2^, MSE, MAE, MBE, and RMSE, respectively, whereas the testing results are 0.96, 30110, 139, -10.63, and 174 for R^2^, MSE, MAE, MBE, and RMSE, respectively. Finally, the validation results are 0.95, 35545, 152, 12.17, and 189 for R^2^, MSE, MAE, MBE, and RMSE, respectively. The overall results indicated that the neural network results with validation dataset are more accurate than the neural network without validation as shown in Table 19.

**Table 19:** Performance of neural network and linear regression compared to real stock market.

Dataset	Predictor	R^2^	MSE	MAE	MBE	RMSE
**Training**	**Linear**	0.94	71485	211	0.00	267
	**Neural Network**	0.98	23620	130	6.22	154
	**Neural Network Validation**	0.98	23620	130	6.22	154
**Testing**	**Linear**	0.85	145897	256	-17.00	382
** **	**Neural Network**	0.95	33176	146	2.23	182
	**Neural Network Validation**	0.96	30110	139	-10.63	174
**Validation**	**Neural Network Validation**	0.95	35545	152	12.17	189

The results indicated that the neural network model is more accurate than the linear regression model in predicting TASI. The neural network error functions also showed that the neural network predictor is reliable and more robust than the linear regression predictor.

**Fig 6:**
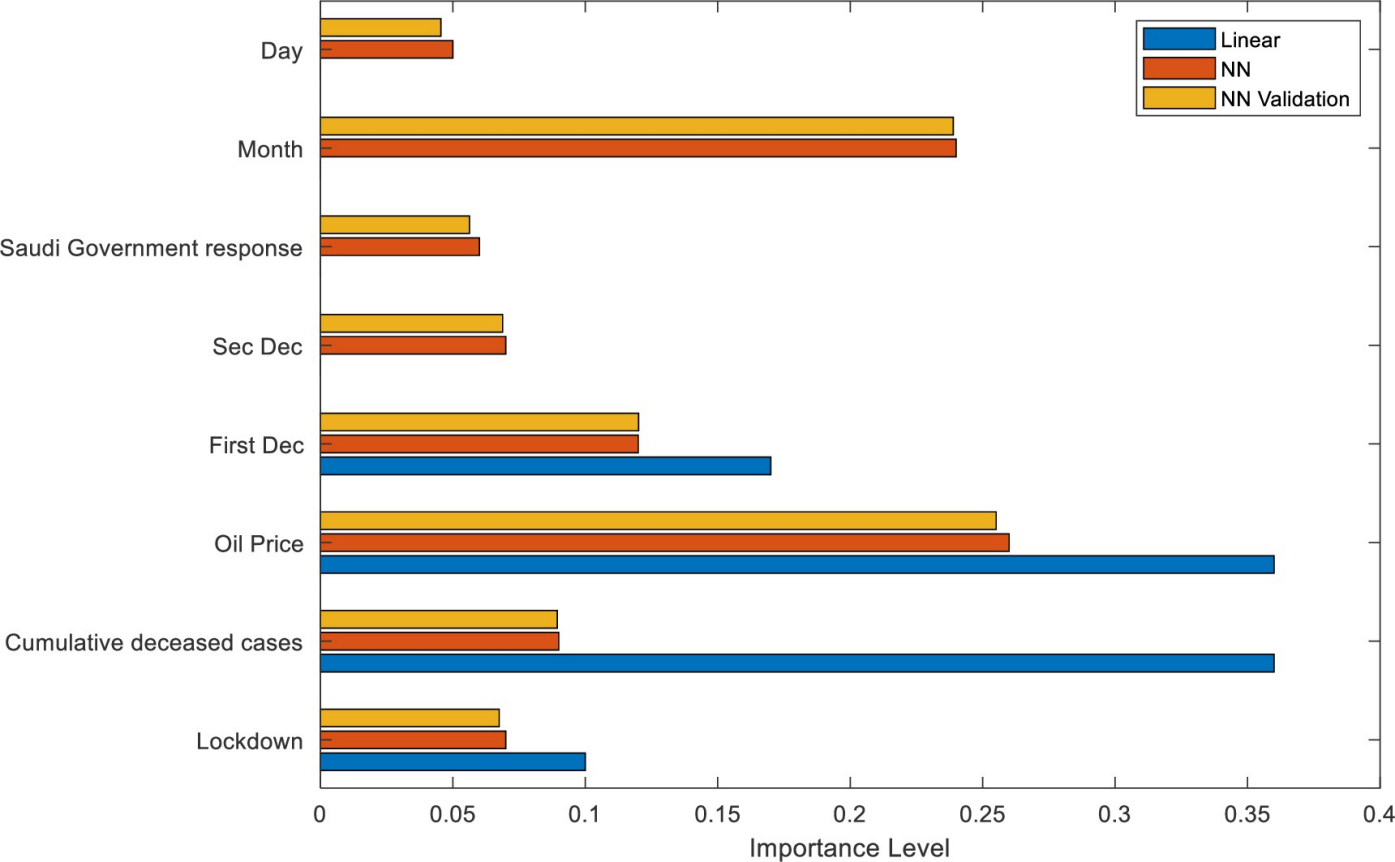
Predictor importance for predicting stock market using neural network and linear predictor.

Moreover, to recognize the importance of the input variables on predicting TASI, the important analysis is considered as shown in Fig 6. The results of the NN and NN validation predictions indicated that the price of oil and the month are among the most important variables in predicting the stock market, where the day variable is considered the least important in the prediction. The level of importance of the remaining variables is sorted from highest to least important as follows: first decrease of Repo and Reverse Repo, cumulative deceased cases, second decrease Repo and Reverse Repo, lockdown, and Saudi Government response. Moreover, the results of the linear predictor showed that the price of oil and first decrease of Repo and Reverse Repo are among the most important variables in predicting TASI, where the day variable is considered the least important variable. The level of importance for the remaining variables is sorted from highest to least important as follows: lockdown, month, and cumulative deceased cases. Finally, the results indicated that even with the validation process, the results would not bring much improvement because the collected dataset is small, making the use of validation not significantly efficient.

The collected results from the important variables of the neural network and linear predictor indicated that oil price is the most important variable in predicting TASI, which is theoretically and practically correct. Saudi Arabia is an oil-based economy with the second largest petroleum reserves, and thus, all macroeconomic factors including the TASI index will be affected by changes in oil prices. However, the findings revealed that the day variable is the least important in both predictors because the day variable is related to the behavioral finance theory in which trading may differ from one day to another because of investors’ moods and emotions. During the pandemic period, the investors were under pressure because of the lockdown and were in continuous fear of the pandemic consequences. Thus, no changes are expected in their investment motivation from one day to another, and thus, the day has a minor effect on investors’ decisions throughout the pandemic era.

The results of the prediction models are lined up with linear regression models and showed that oil prices are the most important variable related to the stock market.

## Conclusions

This study investigated the effects of the global pandemic indicators and oil prices on Tadawul Trading volume and TASI for the period from January 1 to December 2, 2020 using linear regression and neural network predictors (with and without validation process). This paper used the oil prices and the following COVID-19 indicators as independent variables (cumulative deceased cases, lockdown, first^,^ and second decrease Repo and Reverse Repo, Saudi government responses). The analysis was conducted by building two regression models using linear regression models after considering different entering methods (i.e., stepwise, remove and enter methods). Our models have Tadawul trading volume and TASI as dependent variables (one day lagged) and all COVID-19 indicators and oil prices as independent variables. The results showed that R^2^ for trading volume and TASI models are 65.5% and 86% with standard error of 81 and 270, respectively.

According to the analysis of the first model, cumulative deceased cases and the first decrease in Repo and Reverse Repo have a significant positive effect on Tadawul trading volume while oil prices have a significant negative effect on Tadawul trading volume. The second model showed that lockdown and the first decrease in Repo and Reverse Repo have a negative significant effect on TASI. However, cumulative deceased cases and oil prices have a positive effect on TASI.

The results further indicated that neural network models (with and without validation) are more efficient in predicting TASI using all the input variables, and they can be used to predict future TASI values more accurately. In addition, the results of the neural network and linear predictors are in the line with the findings of the linear regression model that indicate the capability of the oil price in affecting the performance of the stock market in Saudi Arabia.

Although COVID-19 has strong effects on all markets and industries, [34] proved that during the COVID-19 period, Asian markets have the highest negative return but this effect started to decrease. [15] also showed that during the COVID-19 pandemic, Asian markets were making better opportunities to diversify the financial risk. All shocks in their first phase were so severe but became less harmful when as time went by, and these markets are expected to regain the confidence of different financial markets and get back on track. [12, 72, 73].

Finally, COVID-19 is one of the worst pandemics we have witnessed in decades, and it has caused stress to the global economy and has had harmful consequences in all aspects. This effect will never be diminished without leveraging people’s awareness of this virus and enhancing government grants and subsidies. According to the Saudi economy and Saudi banking sector, it is highly expected that COVID-19 indicators will be relieved because of the corrective actions of the Saudi government and central bank in addition to Vision 2030 and the transformation program applied at many levels among Saudi sectors.

## Theoretical and practical implications

This research aims to examine the effects of different COVID-19 indicators and oil prices on Tadawul trading volume and TASI and build a neural network model for the factors that can best predict TASI. Hence, this research is theoretically and practically important for investors, academics, and policymakers.

The stock market is one of many macroeconomic indicators linked directly and interactively. Thus, when one indicator changed sharply, others will react and worsen. In our study, we submit extra evidence that stock market indices will react strongly to the changes in macroeconomic factors (i.e. oil prices crash and COVID-19 indicators).

Accordingly, policymakers can benefit from the results by building an early warning system for any unexpected pandemics in the future. Regulators can also benefit from government consequences and decreasing interest rates is one of the best initial choices to protect the whole economy. The importance of Fintech rose considerably during this period, and thus, governments have to focus on such practices to enhance the efficiency and liquidity of the stock market.

The behavioral finance theory holds that stockholders’ investment appetite is affected intensely by many indicators related to stockholders’ emotions and cognitive biases. Existing stockholders within the COVID-19 era were extremely fearful and uncertain because of the consequences of the (i.e., decreasing the interest rates, Saudi government response, lockdown, etc.). Consequently, non-expert investors will respond directly by buying/selling different stocks arbitrarily to avoid the expected losses.

Although random trading will increase the inefficiency of stock markets, professional and sophisticated stockholders can benefit from this situation by specifying the correct timing to enter/exit the market and achieve the maximum capital gains and a sufficient level of liquidity.

## Limitations and further studies

This study has certain limitations and further research is necessary to enhance the results. Future studies can use macroeconomic factors to explore the effects of this pandemic on financial market indices. Further research on the effects of COVID-19 on different financial markets in GCC is necessary. Additional research can also apply long–short term memory and one attention-based neural network to explore the ability to predict the occurrence of any possible pandemic and the stock market indices in different pandemic periods.

## Supporting information

S1 Dataset COVID-19 dataset. (XLSX)
